# Supplementary material for: Multitissue H3K27ac profiling of GTEx samples links epigenomic variation to disease
Source: Nat Genet. 2023 Sep 28;55(10):1665–76. doi: 10.1038/s41588-023-01509-5 (PMC10562256; doi:10.1038/s41588-023-01509-5)
Supplement: Supplementary file 1 — Supplementary Note. [file 41588_2023_1509_MOESM1_ESM.pdf]

nature genetics

Article

https://doi.org/10.1038/s41588-023-01509-5

Multitissue H3K27ac profiling of GTEx samples links epigenomic variation to disease

In the format provided by the authors and unedited

Supplementary Notes

Comparison of H3K27ac profiles across studies

We projected the samples from both this study and the EpiMap reference samples into a lower-dimensional space (**Fig. 1b**). We first extracted -$\log_{10}P$ signals of the tissue-specific AREs mentioned above and removed the bias due to GC content[1]. We then followed the pipeline implemented in Seurat (v3.2.1)[2]: projected both datasets onto the same space by a canonical correlation analysis-based framework (top 30 pairs of canonical variables selected), and carried out dimension reduction by principal component analysis (top 30 PC used) followed by UMAP[3].

We noticed that six samples from reference epigenomes (BSS00080_AORTA, BSS01319_LEG MUSCLE, BSS01332_TRUNK MUSCLE, BSS01463_PSOAS MUSCLE, BSS01201_Lung, BSS01415_LUNG ADENOCARCINOMA) were located far away from other tissue-matched samples (**Fig. 1b**). There are three possible reasons why these samples do not cluster as expected: (1) sample quality issues; (2) technical: the distance between samples may be skewed during the integration processes of the two datasets based on canonical correlation analysis (CCA) in R package Seurat due to unbalanced tissue/cell types between this study (four tissues) and EpiMap (31 tissues); and (3) biological: profiles of bulk samples from different tissues may be similar due to dominant cell types shared. We first confirmed no sample showed any sign of a quality issue (1) based on preprocessing QC metrics and imputation accuracy in a previous report[4]. We then went back to the tissue archetype analysis for EpiMap samples only (**Extended Data Fig. 2f**), where they were studied alone, to test for the technical effect (2) above. We found four of the six above samples (BSS00080_AORTA, BSS01319_LEG MUSCLE, BSS01332_TRUNK MUSCLE, BSS01463_PSOAS MUSCLE) are well clustered with the matched tissue samples before integration with the data from our study, suggesting that the technical effect (2) is the reason why these samples did not cluster as expected. Of the remaining two samples, BSS01201_Lung sample, is clustered with heart samples and showed strong signal in heart archetype, indicating that the biological effect (3) could be the cause of why this sample did not cluster as expected. This may be partially due to the fact that this is a lung sample from a child. Finally, the BSS01415_LUNG ADENOCARCINOMA sample is clustered with other adenocarcinoma samples instead of other lung tumors, which were all lung epithelial carcinoma. Thus, the disparity in clustering for this sample is also likely due to a biological effect (3); it is from a different type of tumor than the other lung tumor samples.

We also used the intersected peak set (250k) between the peak set (282k) in our study and the tissue-specific peak set (405k) identified from Roadmap, and performed correlation analysis (Pearson's correlation coefficient) of H3K27ac signal activity (-$\log_{10}P$) between our samples and samples from EpiMap, and found high correlations (~0.6) between our samples and the biologically-relevant samples from EpiMap (**Extended Data Fig. 1a**).

Deconvolution by estimating tissue-archetype fraction

To account for unwanted variation due to cell fractions, we developed a two-step deconvolution approach to estimate the compositions of each sample: (1) we extracted signals from the reference samples on 282k AREs detected in this study, and decomposed them into 20 tissue-archetype profiles by non-negative matrix factorization implemented in R package NMF (v0.21.0)[5] and used them as the bases (**Extended Data Fig. 2f)**; (2) We then used a linear regression approach to estimate the fraction of each tissue-archetype across our samples based on the bases profiles from the previous step (**Extended Data Fig. 2g)**. Indeed, we found that differences in the top tissue-archetype proportion accounted for 9.8-19.6% of the variation across individuals across the four tissues (brain as an example in **Extended Data Fig. 2h**)

Identify sex-biased ARE and functional annotation

We used R package limma (v3.36.5)[6] to quantify sex-differential signals for ARE activity across samples for each tissue. We included tissue-archetype fractions estimated from deconvolution, known technical and clinical factors that strongly correlated with any of the top PCs (PCC cutoff 0.3), and age as covariates to correct for their effect. We then carried out Benjamini-Hochberg (BH) correction (adjust *P*-value cutoff 0.2) to identify sex-biased AREs, and used Homer to annotate these AREs and identify the closest genes (**Extended Data Fig. 2i**). We calculated enrichment of GO terms for sex-biased ARE by R package rGreat, and followed BH procedure for multiple testing correction (adjust *P*-value<0.1, enrichment fold change>1.5) (**Extended Data Fig. 2i**). We calculated log-transformed fold change and nominal *P*-value for sex-biased genes across tissues

in GTEx with limma voom[7] and sva (v3.28.0)[8], and then compared sex-biased AREs to nearest sex-biased genes (permissive cutoff: nominal *P*>0.01) for coordinated regulations (**Extended Data Fig. 2j**).

Tissue-specificity: eQTL versus haQTL

We focused on the eQTLs both evaluated for tissue specificity by mashR[9] in GTEx v8 and identified as haQTLs in our study. To examine the correlation between eQTL tissue specificity and haQTL tissue specificity, we took the mashR-calibrated eQTLs (GTEx V8) and quantified the tissue-specificity of each eQTL across brain, heart, muscle and lung. For each eQTL in one of our four tissues (defined by local false sign rate (lfsr) < 0.05), we calculated the number of eQTL-sharing tissues with the directionality considered (lfsr < 0.05 & the same effect direction) among the rest of tissues in our study. For the eQTLs that are also haQTLs, we calculated the dependence of eQTL tissue-specificity (number of eQTL-sharing tissues) on haQTL tissue-specificity (Type 1, 2, and 3 for the increasing tissue-specific gARE types: haQTL-Shared, haQTL-specific, and ARE-specific, respectively) by linear regression (**Extended Data Fig. 3i**).

Colocalization between GWAS, haQTL and eQTL signals

We used coloc (v3.2-1)[10], a Bayesian framework, to estimate the pairwise posterior probability (PP4) of causal SNPs colocalized among GWAS, haQTL, and eQTL signals based on summary statistics. We applied a more permissive cutoff to define gAREs (empirical *P*-value cutoff 0.05, 11,942, 8240, 9678, 5375 gAREs for brain, heart, muscle, and lung, respectively) for colocalization analysis given that: (1) our results on haQTL sharing (**Fig. 3c**) demonstrate that the haQTL effect is still reliable even for a relatively weak nominal *P*-value (0.01-0.1); (2) coloc could sensitively and reliably detect the colocalization events between haQTL and GWAS or eQTL, even when haQTLs effect is relatively weak; and (3) enrichment analyses on FMeQTL-proximal gAREs (**Fig. 5a**) and FM-eQTLs interrupting TF binding sites in permissive gAREs (**Extended Data Fig. 5b**) both demonstrated that gAREs defined by empirical *P*-value<0.05 show strong enrichment over the background.

The eQTL summary statistics are from the haQTL-matched tissues from GTEx v8. We carried out GWAS-haQTL and haQTL-eQTL colocalization analyses for SNPs within 100kb to each gARE tested. We only included gAREs with significant GWAS signals (GWAS nominal $P<1\times10^{-5}$) nearby (100kb window), and used PP4≥0.5 to define GWAS-haQTL-colocalized gAREs. We carried out GWAS-eQTL colocalization for those gARE loci with significant GWAS signals (GWAS nominal $P<1\times10^{-5}$) and significant eQTL signals in matched tissue by GTEx nearby (100kb window) (**Fig. 4b, Extended Data Fig. 4a**). To identify eQTL-missing GWAS-haQTL-colocalized gAREs, we carried out GWAS-eQTL colocalization for each GWAS-haQTL-colocalized gAREs locus (100kb window) after removing the MHC locus, and defined an eQTL-missing event as PP4<0.1 for all eGenes around the tested region. Colocalization results are visualized by R package sushi (v1.18.0)[11] (**Fig. 4c, d, and f**).

For colocalization analysis with SuSiE (**Extended Data Fig. S4b**), we used R package susieR (v0.12.27, R-4.0)[12] to identify the independent signals for both GWAS and eQTL at each locus, then run colocalization with R coloc package for each pair of independent signals, and reported the largest PP4 among all the pairs as the final result.

We showed a global picture of sharing and specificity of haQTL and eQTL colocalization with GWAS signals in the brain across multiple traits. eQTL-specific GWAS colocalization loci (GWAS-eQTL coloc PP4≥0.5 and GWAS-haQTL coloc PP4≤0.1) outnumber haQTL-specific GWAS colocalization loci (GWAS-haQTL coloc PP4≥0.5 and GWAS-eQTL coloc PP4≤0.1) with a median ratio of 17.4:1 (**Extended Data Fig. 4a**). This result is expected, given that (1) the majority of the non-coding regulatory elements impact phenotype through gene expression, therefore eQTLs more directly explain phenotypic variation than haQTLs do by nature; and (2) eQTLs in GTEx are discovered with higher power than haQTL in our cohort (175, 706, 386, and 515 samples for eQTL mapping vs 109, 81, 89, 53 samples for haQTL mapping in brain, muscle, heart and lung, respectively). Collectively, the colocalization across haQTL, eQTL, and GWAS revealed different modes of regulation, suggesting the complexity of regulatory circuits in different loci and genetic traits, and the necessity to include epigenomic QTL studies.

bulk-eQTL-missing GWAS loci

In our analyses, we identified "bulk-eQTL-missing GWAS loci", defined as GWAS signals colocalized with haQTL signals but missed by GTEx bulk-eQTL signals (**Fig. 4d-e**). Most of them could be captured by cell-type-level eQTL signals based on colocalization analyses with eQTLs from an sn-RNAseq study (**Extended Data Fig. 4c**). However, we should also note that there are still three of these loci that are captured by haQTL but missed by either bulk or cell-type-level eQTL (in red box). Furthermore, to detect robust loci missed by eQTL signal, we used a stringent coloc cutoff for GWAS-haQTL colocalization (PP4<0.5) and a permissive one for GWAS-eQTL (PP4<0.1). If we use the same cutoff of 0.5 for both analyses, there are 11 GWAS loci captured by haQTL but missed by both bulk and cell-type-level eQTLs, even though the sample size of haQTL detection (109) is smaller than bulk (175) and cell-type eQTL (192). One possible explanation for the weak GWAS-eQTL' colocalization signal for both types of eQTLs is that they have been detected in non-disease-relevant conditions. Thus, eQTL regulation in cell type-specific and condition-specific manners may jointly account for the missing eQTL signal at GWAS loci we observed. It also partially explains the "missing regulation", a potential bias to infer disease target genes based on eQTL for GWAS signal described in a previous report[13]. These results also indicate that bulk epigenomic variation may capture the impact of genetic variants (as shown here by haQTL effects) with a greater power than bulk eQTL for certain loci where the effect may become visible only in specific cell types or specific conditions.

Mendelian Randomization

We applied Mendelian Randomization, specifically MR-Egger implemented in R package MendelianRandomization (v0.4.1)[14], to infer the causal effect of ARE activity on either gene expression or phenotypic risk based on haQTL/eQTL/GWAS summary statistics. We filtered SNPs requiring its haQTL effect (adjusted haQTL *P*-value cutoff of 0.2, by BH test carried out for all SNPs in the locus) and pruned by r^2 estimated by plink[15] (cutoff 0.6). We reported those ARE loci potentially causal to phenotypic risk with adjust *P* <0.2 after BH multiple test correction, and reported the adjusted *P-value* for ARE's impact on gene expression as one of the gARE-gene gLink scores.

QTL-proximal gARE enrichment

We defined the QTL-proximal region as the region with an eQTL or an FM-eQTL located within 2kb from its center. We used a 2kb window to link eQTL to H3K27ac peaks to capture the 'correct' dip of the peak that might be responsible for the signal[16], or the correct 'set of dips' in the case of multiple driver enhancers and potentially multiple variants in the same LD block[17]. Our enrichment analysis showed that thresholds up to 2kb show the highest enrichment, after which the enrichment curves flatten out for fine-mapped eQTLs (**Extended Data Fig. 5a**), thus a 2kb window is appropriate to maximize sensitivity.

We used eQTL from GTEx v8, FM-eQTLs inferred by CAVIAR[18] provided in GTEx v8, and gAREs with a permissive cutoff (empirical *P*-value<0.05). We generated four types of genomic regions as background with BEDTools[19]: (1) AREs: all AREs in a specific tissue; (2) ARE flanking regions: regions between two adjacent AREs; (3) AREs from other tissues: AREs merged from the rest of the tissues with AREs from the target tissue removed; and (4) shuffled genomic regions, with the same number of genomic regions of the same size randomly picked from the genome (**Fig. 5a**). We also tested the enrichment of FMeQTLs interrupting a TF binding site (motif scan results for ARE modules above) localized in gAREs compared to all FMeQTLs by proportion test (**Extended Data Fig. 5b**).

gLink scores

We only considered gAREs (empirical *P*-value<0.05) located between 2kb and 1Mb of an eGene transcription start site (TSS) as potential paired enhancers. gLink scores identify linkages from two perspectives on how eQTLs may regulate gene expression related to an ARE: (1) the genetic variant functions through a proximal ARE, but it may not necessarily regulate the ARE's activity; (2) the genetic variant regulates both an ARE and a gene. gLink scores include six scores: (1) gARE-dist-to-FMeQTL: the shortest distance between FM-eQTLs of an eGene to a specific gARE; (2) gARE-proximal-eQTL: the smallest nominal *P*-value of eQTLs of an eGene to a proximal gARE (within 2kb); (3) coloc-PP4: the coloc PP4 between haQTL of a gARE and eQTL of an eGene; (4) coloc-PP4/3: the ratio of PP4 to PP3, and set to 0 if coloc-PP4<0.1; (5) MR: adjusted *P*-value (BH) for MR-Egger test depicted in the section of Mendelian Randomization; (6) ExpPGS-gARE-corr.: we first generated expression polygenic score by elastic net model implemented in R package glmnet[20] trained with

GTEx individual data (alpha set as 0.5, lambda is optimized from cross validation), and then calculated adjusted *P*-value (BH) of the correlation between expression polygenic score and gARE activity across individuals after correcting for the covariates included in haQTL mapping.

We also standardized each of the six gLink scores so that they are comparable. For a gLink score $s_{i,j}$, denoting the candidate ARE-gene pair i from the jth gLink score, we calculated the standardized score as the precision with $s_{i,j}$ as cutoff on the PRC for j^{th} gLink score with ABC score as the benchmark dataset. We then used the maximum transformed score among six gLink scores as a unified gLink score for each candidate linking. We generated a high-confidence linking set for each tissue based on the cutoff corresponding to the original gLink score cutoff of 2kb for gARE-dist-to-FMeQTL due to consistent performance of the score (**Fig. 5c**) and the fact that 2kb is at the elbow of the enrichment curve (**Extended Data Fig.5a**). We finally prioritized GWAS-haQTL-colocalized gARE-gene circuits based on the high-confidence linking sets for schizophrenia after removing the MHC locus (**Fig. 6c and Extended Data Fig. 6e**), and visualized with R package ComplexHeatmap (v2.4.3, R-4.0)[21].

We are also aware of the limitations of this unified score system: (1) ABC may not be the ideal benchmark dataset; and (2) a unified score may ignore the fact that the performance of each score at specific loci depends on the power of different QTLs which are used to derive gLink scores. Approach 1 gLink scores (*gARE-dist-to-FMeQTL* and *gARE-proximal-eQTL*) rely on eQTL power while approach 2 gLink scores (*coloc-PP4, coloc-PP4/3, MR,* and *ExpPGS-gARE-corr.*) rely on both eQTL and haQTL powers. Thus, we highly recommend that users select gLink scores or build a unified score based on their own data and context.

Comparing gLink scores with other scores

We included each eGene reported in GTEx v8 and gAREs (empirical *P*-value<0.05) in its *cis*-window (2kb-1Mb from TSS) as candidate linking pairs for gLink scores and other scores to compare. We first compared the performances of gLink scores by taking each of them as the benchmark dataset (pairs with the top 1% score as the positive set and the rest as the negative set), and calculated AUPRC (**Extended Data Fig. 5d**) with R package PRROC[22]. We also generated EpiMap linking (with our AREs) and ABC scores (with our AREs and H3K27ac activity) in each tissue, and used each of them as the benchmark dataset to evaluate gLink scores (pairs with top 5% as positive set and the rest as negative set) by AUPRC with R package PRROC (v1.3.1)[22] (**Fig. 5c and Extended Data Fig. 5e**).

We compared the proportions of GWAS-haQTL-colocalized gAREs (PP4 ≥0.1) linked to a target gene prioritized by different linking scores (top 10% links for each score) relative to the background, defined as the proportion of all the gAREs with candidate gARE-gene pairs (gARE-TSS distance within 2kb-1Mb) that are captured by GWAS-haQTL colocalization (PP4 ≥0.1) (**Fig. 6a** and **Extended Data Fig. 6a** top two rows). We confirmed gLink scores with higher enrichments for brain-related traits after changing the linking cutoff to top 20% links (**Extended Data Fig. 6a** bottom two rows). gLink scores perform better in identifying disease target genes supported by GWAS-eQTL-colocalization (**Fig. 6b**). It is as expected since eQTLs were used as one of the information sources in the gLink scores. We also compared the target genes (top 10% links) to genes predicted in a previous study[23] for each of the brain-related traits (**Extended Data Fig. 6b**). The target genes predicted for these traits were also used as validation sets for another SNP-gene linking prediction paper[24], and can be downloaded from here (https://alkesgroup.broadinstitute.org/cS2G/critical_gene_sets/).

In comparison with the other state-of-the-art approaches, we found that the gLink scores are consistent with the ABC score, and less consistent with the EpiMap linking score, similar to the discrepancies among different methods reported previously[24,25]. The discrepancy between gLink and EpiMap linking scores is as expected: gLink scores are based on inter-individual variation associated with genetics in the same tissue, while EpiMap linking scores are based on inter-tissue/cell type variation. Thus, the gLink scores may capture a different aspect of the enhancer-gene regulatory network and provide a complementary perspective compared to other scores. gLink scores also show stronger enrichment for GWAS-haQTL-colocalized gAREs compared to gene-ARE distance, EpiMap, and ABC scores. This could be because gLink scores tend to capture gARE-proximal genetic signals associated with gene expression, which are more likely to be associated with GWAS signals.

Altogether, gLink scores are able to: (1) capture genetically-driven links that may be missed by other approaches; and (2) track the impact of disease-associated genetic variants on both enhancers and genes in a tissue-specific manner, which is missed by GWAS-eQTL colocalization

Extended Data Figure Caption

Extended Data Figure 1. Correlation of H3K27ac profiles between samples in this study and those from the reference epigenomes. Each column represents a sample in our study with tissue name on the top, and each row represents a sample from the reference epigenomes; for each sample in our study, the top five highly correlated reference samples are labeled with "*"; orange, red, green and blue boxes indicate tissue-matched pairs between our data and the reference data.

Extended Data Figure 2. Tissue specificity of AREs and functional annotations of ARE modules. a, ARE tissue-specificity and sharing across brain, heart, muscle, and lung. The Venn diagram shows the numbers and proportions of AREs for different combinations of tissue-sharing across four tissues. **b**, 282k AREs identified in this study form 1413 submodules from 127 modules based on coactivity across 240 reference epigenomes. Upper panel: ARE activity of 1413 submodules (by column) across samples (by row) in our study; orange, red, green, and blue boxes showing tissue-specific modules for brain, heart, muscle, and lung, respectively; sex and tissue information are on the right. Lower panel: ARE activity of 1413 submodules in the reference epigenomes; sample clusters annotated on the right. **c**, GO biological processes enrichment for 127 ARE modules. Each row represents a GO term and each column represents an ARE module with ARE group labeled at the bottom; red, green, orange, and blue boxes indicate the enrichment for G3, G9, G12, and lung-specific modules.

Extended Data Figure 3. TF motif enrichment of ARE modules and ARE detection power. a, Motif enrichment for enhancer modules. Each row denotes a TF family, represented by the TF labeled on the right having the strongest odds ratio across modules; each column represents an ARE module with ARE group labeled at the bottom; red, green, orange, blue, and purple boxes indicate enrichment for G3, G9, G12, and lung-specific modules. **b,** Comparison of ARE detection rates between *Newly-detected* ARE (G14) and the other groups. X-axis shows the number of brain samples randomly selected for each experiment; y-axis shows the proportion of AREs detected from each experiment; colors denote which groups AREs are from; n=10 independent times of sampling for each box.

Extended Data Figure 4. Tissue-archetype fraction estimation. a, Deconvolution step 1. The heatmap shows the correlation between the profiles of tissue-archetype (by column) and the profiles for the reference samples (by row) with strong tissue-archetype specific patterns. Typical sample names are shown on the right, four samples that are not clustered with other tissue-matched samples in **Figure 1b** and mentioned in the section of "Comparison of H3K27ac profiles across studies" are labeled on the left. **b,** Deconvolution step 2. The heatmap shows the fraction of each tissue-archetype (by column) estimated for samples (by row) in each of our tissues, with the primary tissue-archetypes indicated by gray boxes.

Extended Data Figure 5. Sex-biased ARE identification. a, Comparison between principal components (PCs) and covariates including estimated tissue-archetype fractions and known factors for brain samples. Top left panel: heatmap shows the correlation between PCs (by column) and known factors (by row); Top right panel: percentage of variation (x-axis) explained by the covariates (by row), with red highlight for the primary tissue-archetype identified in **Extended Data Fig. 4a**; Bottom panel: the percentage of variation (y-axis) explained by each PC for brain samples (by column). **b,** Sex-biased AREs, activity pattern and annotations. Left panel: enrichment of sex-biased genes from matched GTEx tissue (by column) in genes closest to sex-biased AREs identified from this study; * denotes strong enrichment (adjusted P <0.1, two-sided Fisher's exact test, BH correction across multiple tissues tested, shown in **Supplementary Table 2**); middle panel: sex-biased ARE activity of each sample (by column) in each tissue, with top 5 sex-biased genes closest to any sex-biased ARE labeled on the right; right panel: GO biological processes enriched for genes near sex-biased AREs; purple and blue colors represent female-biased and male-biased genes and terms, respectively, for middle and right panels. **c**, Coordinated regulation of ARE activity and gene expression by sex. Left panel**:** ARE activity for the sex-biased AREs in heart samples; right panel: sex-differential signal for the genes closest to the sex-biased AREs in heart; Boxes = 25%-75% percentile (i.e. inter-quartile range; IQR); line = median;

whiskers = 1.5 IQR.

Extended Data Figure 6. Identification of haQTLs. a, Comparison between Peer factors (by column) and covariates (by row) including estimated tissue-archetype fraction and known factors for brain. **b**, Power analysis for haQTL mapping. Colors indicate different minor allele frequencies, and vertical dashed lines denote current sample sizes for each tissue. **c**, Saturation analysis for haQTL detection in the brain based on down-sampling. For each sample size, 10 randomly down-sampling were performed. The x-axis denotes sample sizes after downsampling, while the y-axis denotes the detection rate of the haQTLs from the downsampled data relative to the haQTLs detected from the full data. The boxes show the 25th–75th percentile; the lines show the median; the whiskers show 1.5 × IQR. **d**, Distribution of the genomic distance between a gARE and its lead haQTL for brain.

Extended Data Figure 7. haQTL tissue-specificity. a, Quantification of haQTL pairwise tissue-sharing based on directionality consistency. The x-axes show the $-\log_{10}$(*P*-value) of haQTLs in the replication tissue, separated by positive effect (right half-plane) and negative effect (left half-plane) in the discovery tissue; the y-axes show the haQTL effect sizes in the replication tissue; haQTLs *P*-values shown in panels **a-d** are all nominal *P*-values based on linear regression (two-sided test). **b,** Quantification of haQTL pairwise tissue-sharing based on similarity of effect size. The x-axes show the haQTL effect sizes in the discovery tissue, and the y-axes show the haQTL effect sizes in the replication tissue. **c,** The effect size similarity, defined as the coefficient of effect size between the replication tissue and discovery tissue, increases as the *P*-value significance increases in the replication tissue; the centers represent the estimated coefficient, and the error bars denote standard errors of the estimation. **d,** Identification of Type-I gAREs (haQTL-Shared) based on the nominal *P*-values in the replication tissue. The black curve shows directionality consistency (the y-axis on the left) of gAREs passing the *P*-value threshold on the x-axis; the green curve shows the count of gAREs (the y-axis on the right) passing the nominal *P*-value threshold on the x-axis; the nominal *P*-value threshold was set to 0.02 in the replication tissue to define a Type-I gARE (haQTL-Shared) between the discovery and replication tissues, which makes the directionality consistency between the two tissues be over 95%. **e,** gARE type explains eQTL tissue specificity. Different panels represent results for each tissue; x-axis represents the different type of gARE with increasing tissue specificity; y-axis represents eQTL tissue-specificity, the number of eQTL-sharing tissues; *P*-values testing the dependence of eQTL tissue-specificity on gARE tissue-specificity (linear regression, two-sided) are shown on top.

Extended Data Figure 8. GWAS-haQTL and -eQTL colocalization. a, GWAS-haQTL vs. GWAS-eQTL colocalization over 1694 brain gARE loci with significant brain eQTL. The x-axis shows different GWAS traits, and the y-axis denotes the counts of GWAS-haQTL or GWAS-eQTL colocalization events from three types: shared colocalization (in red, both GWAS-eQTL and GWAS-haQTL coloc PP4 ≥0.1, at least one of them ≥0.5), haQTL-specific colocalization (in green, GWAS-haQTL coloc PP4 ≥0.5 and GWAS-eQTL coloc PP4 <0.1), and eQTL-specific colocalization (in blue, GWAS-eQTL coloc PP4 ≥0.5 and GWAS-haQTL coloc PP4 <0.1). **b,** A comparison between schizophrenia GWAS-eQTL colocalization analyses with/without SuSiE. The heatmap shows the coloc PP4 for each gARE locus (by row, labeled on the right, hg38 coordinates) with each method (by column, labeled on top). * marks blocks missing colocalization signal (coloc PP4 <0.1), while arrow points out the only loci missed by coloc and captured by coloc with SuSiE. **c,** A comparison between schizophrenia GWAS-eQTL colocalization analyses using brain bulk and cell-type-level eQTL. The heatmap shows the coloc PP4 for each gARE locus (by row, labeled on the right, hg38 coordinates) based on bulk haQTL, bulk eQTL, and eQTL from eight cell types in the brain (by column). * marks blocks with weak colocalization signal (coloc PP4 ≥0.1), while ** marks blocks with strong colocalization signal (coloc PP4 ≥0.5). Orange dot on the right marks the loci only captured by cell-type-level eQTL (coloc PP4 ≥0.1), while green dot marks the loci missed by both types of eQTLs with stringent cutoff (coloc PP4 <0.5). Three gARE loci with strong GWAS-haQTL

colocalization signals (coloc PP4≥0.5) and missed by both types of eQTLs even at the permissive cutoff (coloc PP4 <0.1) are shown in red box.

Extended Data Figure 9. Properties of gLink scores. a, eQTL/FM-eQTL proximal enrichment of genomic regions at different bins of SNP-region distance. The plots show the fold enrichment of QTL-proximal regions in target regions (in different color) over shuffled genomic regions for eQTL (left) and FM-eQTL (right) for each tissue. The y-axis shows the enrichment, while the x-axis shows different SNP-region bins (0-250bp, 250bp-500bp, 0.5-1, 1-1.5, 1.5-2, 2-2.5, 2.5-3, 3-3.5, 3.5-4, 4-4.5, 4.5-5kb). The blue dashed line marks 2kb which we chose as the cutoff to define the proximity. **b,** Proximal gAREs enrich FMeQTLs interrupting TF binding sites. The y-axis shows the proportion of FMeQTLs that interrupt TF binding sites from **Extended Data Fig. 2c**; orange bars represent the FMeQTLs located in gAREs, and gray bars represent FMeQTLs in all AREs. **c,** FMeQTL-proximal gAREs do not guarantee shared genetic regulation between gene and ARE. Each point: gARE-gene pair based on *gARE-dist-to-FMeQTL* score (distance cutoff of 2kb); x-axes denote the shared genetic regulation at the SNP-level (nominal *P* for haQTL, linear regression, two-sided); y-axes denote the shared genetic regulation at the locus-level; Red circles indicate pairs without evidence of shared genetic regulation at both the locus and SNP levels. Percentage of FmeQTL-proximal gAREs without shared genetic regulation in each tissue shown at the top of each graph. **d,** Performances of gLink scores with one of them as the benchmark dataset (AUPRC). The heatmaps show AUPRC for each gLink score (by row) with one of scores as the benchmark dataset (by column) for each tissue; red dashed boxes indicate results for gLink scores from approach 2, showing higher consistency between these scores. **e,** Performance of gLink scores with EpiMap score as the benchmark dataset. We showed PRC of gLink scores for each tissue, which is barely higher than that of background.

Extended Data Figure 10. gLink scores prioritize gARE-gene circuits for diseases and traits. a, Enrichment of GWAS-haQTL-colocalized gAREs in the gAREs with predicted links for heart- and lung-related traits. Figure format as in Fig. 6a; * shows the significance levels (one-sided proportion test); HPT, hypertension; CH2, MAGNETIC_CH2.DB.ratio; HDLC, MAGNETIC_HDL.C; IDL, MAGNETIC_IDL.TG; ATH, asthma; FEV1, volume that has been exhaled at the end of the first second of forced expiration; FVC, Forced Vital Capacity; PEF, Peak expiratory flow; UKB, UK biobank; UKBS, self reported traits from UK biobank. **b**, Target genes inferred compared to the predictions from Weeks *et al*. The x-axes denote the percentage of genes inferred from each approach (by row) overlapping with the disease genes from a previous report for each disease (by panel); * shows the significance levels (two-sided Fisher's exact test). INSOMN, insomnia. **c,** Comparison between target genes from different linking scores. The heatmap shows the mean of the similarity of target genes (Jaccard index) across 10 brain-related traits in Fig. 6a for each pair of linking scores. **d,** Distribution of the distance between a GWAS-haQTL-colocalized gARE and its predicted target gene by each linking score for the 10 brain-related traits in **Fig. 6a**. Boxes = 25%-75% percentile (i.e. inter-quartile range; IQR); line = median; whiskers = 1.5 IQR; number of gARE-gene pairs shown. **e,** Schizophrenia GWAS-haQTL-colocalized gARE-gene circuits only in muscle or heart. Left panel: The heatmap shows the genetic evidence of association between target gene and schizophrenia for each gARE-gene circuit (by row) in each tissue (by column); genomic position of ARE and ARE group shown on the left; for each cell, upper triangle shows evidence based on GWAS-eQTL colocalization (PP4), and lower triangle shows number of gLink scores that connect GWAS-haQTL-colocalized gARE to the same gene; genes in red text on right side of heatmap identified as fibroblast subtype marker genes from a brain vasculature sc-RNA-seq study. Right panel: upper, UMAP result of sc-RNA-seq profiles with cell subtype labeled from a brain vasculature study, and lower, *WBP1L* expression level marked in the UMAP. **f,** Schizophrenia GWAS-eQTL colocalization for gAREs loci from panel **e**. The heatmap shows GWAS-eQTL PP4 across 13 brain-related tissues and 7 muscle/heart-related tissues (by column) for each gARE (by row); ARE group and GWAS-haQTL colocalization are annotated on left.

Reference

1. Teng, M. & Irizarry, R. A. Accounting for GC-content bias reduces systematic errors and batch effects in ChIP-seq data. *Genome Res.* **27**, 1930–1938 (2017).

2. Stuart, T. *et al.* Comprehensive Integration of Single-Cell Data. *Cell* vol. 177 1888–1902.e21 Preprint at https://doi.org/10.1016/j.cell.2019.05.031 (2019).
3. McInnes, L., Healy, J. & Melville, J. UMAP: Uniform Manifold Approximation and Projection for Dimension Reduction. *arXiv [stat.ML]* (2018).
4. Boix, C. A., James, B. T., Park, Y. P., Meuleman, W. & Kellis, M. Regulatory genomic circuitry of human disease loci by integrative epigenomics. *Nature* **590**, 300–307 (2021).
5. Gaujoux, R. & Seoighe, C. A flexible R package for nonnegative matrix factorization. *BMC Bioinformatics* **11**, 367 (2010).
6. Ritchie, M. E. *et al.* limma powers differential expression analyses for RNA-sequencing and microarray studies. *Nucleic Acids Res.* **43**, e47 (2015).
7. Law, C. W., Chen, Y., Shi, W. & Smyth, G. K. voom: precision weights unlock linear model analysis tools for RNA-seq read counts. *Genome Biol.* **15**, 1–17 (2014).
8. Leek, J. T., Evan Johnson, W., Parker, H. S., Jaffe, A. E. & Storey, J. D. The sva package for removing batch effects and other unwanted variation in high-throughput experiments. *Bioinformatics* **28**, 882 (2012).
9. Urbut, S. M., Wang, G., Carbonetto, P. & Stephens, M. Flexible statistical methods for estimating and testing effects in genomic studies with multiple conditions. *Nat. Genet.* **51**, 187–195 (2019).
10. Giambartolomei, C. *et al.* Bayesian test for colocalisation between pairs of genetic association studies using summary statistics. *PLoS Genet.* **10**, e1004383 (2014).
11. Phanstiel, D. H., Boyle, A. P., Araya, C. L. & Snyder, M. P. Sushi.R: flexible, quantitative and integrative genomic visualizations for publication-quality multi-panel figures. *Bioinformatics* **30**, 2808–2810 (2014).
12. Wang, G., Sarkar, A., Carbonetto, P. & Stephens, M. A simple new approach to variable selection in regression, with application to genetic fine mapping. *Journal of the Royal Statistical Society: Series B (Statistical Methodology)* vol. 82 1273–1300 Preprint at https://doi.org/10.1111/rssb.12388 (2020).
13. Connally, N. *et al.* The missing link between genetic association and regulatory function. *Elife* **11**, (2022).
14. Yavorska, O. O. & Burgess, S. MendelianRandomization: an R package for performing Mendelian randomization analyses using summarized data. *International Journal of Epidemiology* vol. 46 1734–1739 Preprint at https://doi.org/10.1093/ije/dyx034 (2017).
15. Purcell, S. *et al.* PLINK: a tool set for whole-genome association and population-based linkage analyses.

Am. J. Hum. Genet. **81**, 559–575 (2007).

16. Ernst, J. *et al.* Genome-scale high-resolution mapping of activating and repressive nucleotides in regulatory regions. *Nat. Biotechnol.* **34**, 1180–1190 (2016).
17. Corradin, O. *et al.* Combinatorial effects of multiple enhancer variants in linkage disequilibrium dictate levels of gene expression to confer susceptibility to common traits. *Genome Res.* (2013) doi:10.1101/gr.164079.113.
18. Hormozdiari, F., Kostem, E., Kang, E. Y., Pasaniuc, B. & Eskin, E. Identifying causal variants at loci with multiple signals of association. *Genetics* **198**, 497–508 (2014).
19. Quinlan, A. R. & Hall, I. M. BEDTools: a flexible suite of utilities for comparing genomic features. *Bioinformatics* **26**, 841–842 (2010).
20. Friedman, J., Hastie, T. & Tibshirani, R. Regularization Paths for Generalized Linear Models via Coordinate Descent. *J. Stat. Softw.* **33**, 1–22 (2010).
21. Gu, Z., Eils, R. & Schlesner, M. Complex heatmaps reveal patterns and correlations in multidimensional genomic data. *Bioinformatics* **32**, 2847–2849 (2016).
22. Grau, J., Grosse, I. & Keilwagen, J. PRROC: computing and visualizing precision-recall and receiver operating characteristic curves in R. *Bioinformatics* vol. 31 2595–2597 Preprint at https://doi.org/10.1093/bioinformatics/btv153 (2015).
23. Weeks, E. M. *et al.* Leveraging polygenic enrichments of gene features to predict genes underlying complex traits and diseases. *medRxiv* 2020.09.08.20190561 (2020) doi:10.1101/2020.09.08.20190561.
24. Gazal, S. *et al.* Combining SNP-to-gene linking strategies to identify disease genes and assess disease omnigenicity. *Nat. Genet.* **54**, 827–836 (2022).
25. Moore, J. E., Pratt, H. E., Purcaro, M. J. & Weng, Z. A curated benchmark of enhancer-gene interactions for evaluating enhancer-target gene prediction methods. *Genome Biol.* **21**, 17 (2020).
