# Supplementary material for: Multitissue H3K27ac profiling of GTEx samples links epigenomic variation to disease
Source: Nat Genet. 2023 Sep 28;55(10):1665–76. doi: 10.1038/s41588-023-01509-5 (PMC10562256; doi:10.1038/s41588-023-01509-5)
Supplement: Supplementary file 2 — Reporting Summary [file 41588_2023_1509_MOESM2_ESM.pdf]

nature portfolio

Corresponding author(s): Manolis Kellis

Last updated by author(s): Jun 1, 2023

Reporting Summary

Nature Portfolio wishes to improve the reproducibility of the work that we publish. This form provides structure for consistency and transparency in reporting. For further information on Nature Portfolio policies, see our Editorial Policies and the Editorial Policy Checklist.

Statistics

For all statistical analyses, confirm that the following items are present in the figure legend, table legend, main text, or Methods section.

n/a	Confirmed	
☐	☒	The exact sample size (*n*) for each experimental group/condition, given as a discrete number and unit of measurement
☐	☒	A statement on whether measurements were taken from distinct samples or whether the same sample was measured repeatedly
☐	☒	The statistical test(s) used AND whether they are one- or two-sided<br>*Only common tests should be described solely by name; describe more complex techniques in the Methods section.*
☐	☒	A description of all covariates tested
☐	☒	A description of any assumptions or corrections, such as tests of normality and adjustment for multiple comparisons
☐	☒	A full description of the statistical parameters including central tendency (e.g. means) or other basic estimates (e.g. regression coefficient) AND variation (e.g. standard deviation) or associated estimates of uncertainty (e.g. confidence intervals)
☐	☒	For null hypothesis testing, the test statistic (e.g. F, t, r) with confidence intervals, effect sizes, degrees of freedom and P value noted<br>*Give P values as exact values whenever suitable.*
☒	☐	For Bayesian analysis, information on the choice of priors and Markov chain Monte Carlo settings
☒	☐	For hierarchical and complex designs, identification of the appropriate level for tests and full reporting of outcomes
☐	☒	Estimates of effect sizes (e.g. Cohen's *d*, Pearson's *r*), indicating how they were calculated

Our web collection on statistics for biologists contains articles on many of the points above.

Software and code

Policy information about availability of computer code

Data collection	The H3K27ac ChIP-seq profiling sequencing data was generated at the Genomics Platform and the Broad Technology Labs (BTL) using the Illumina HiSeq 2500 sequencer. Raw sequencing data was aligned and processed using bwa (v0.5.9).
Data analysis	We used samtools (v1.3.1), MACS2 (v2.1.1) for peak calling, bedtools (v2.26.0) for peak processing, FastQTL (v2.184) for QTL identification, LDSC (v1.0.0) for GWAS inheritability analysis, HOMER (v4.11.1) for motif analysis. We mainly used R (v3.6.0) unless specified, and used following R packages in the analysis: Seurat (v3.2.1), flexclust (v1.4-0), annotatr (v1.6.0), rGREAT(v1.2.1), NMF (v0.21.0), limma (v3.36.5), sva (v3.28.0), Peer (v1.0), MR (v0.4.1), coloc (v3.2-1), susieR (v0.12.27, R-4.0), Sushi (v1.18.0), powereQTL (v0.3.4), PRROC (v1.3.1), ComplexHeatmap (v2.4.3, R-4.0) .

For manuscripts utilizing custom algorithms or software that are central to the research but not yet described in published literature, software must be made available to editors and reviewers. We strongly encourage code deposition in a community repository (e.g. GitHub). See the Nature Portfolio guidelines for submitting code & software for further information.

March 2021

Data

Policy information about availability of data

All manuscripts must include a data availability statement. This statement should provide the following information, where applicable:

- Accession codes, unique identifiers, or web links for publicly available datasets
- A description of any restrictions on data availability
- For clinical datasets or third party data, please ensure that the statement adheres to our policy

Supplementary info and datasets are linked from http://compbio.mit.edu/eGTEx-H3K27ac/. H3K27ac ChIP-seq profiles from this study are available on dbGaP with accession number phs000424.v8.p2. Additionally, the data can be accessed via AnVIL with authentication: https://anvil.terra.bio/#workspaces/anvil-datastorage/AnVIL_GTEx_V8_hg38. Since the raw sequencing data with genetic information are protected, application and authentication are needed before accessing the data. All non-protected data of H3K27ac ChIP-seq can be visualized via the GTEx Portal (www.gtexportal.org) as part of eGTEx v8.

Human research participants

Policy information about studies involving human research participants and Sex and Gender in Research.

Reporting on sex and gender	We added sex and age in the supplementary table 1 as part of meta information
Population characteristics	This study does not involved human research participants, since samples are postmortem from the GTEx cohort and deidentified.
Recruitment	*Describe how participants were recruited. Outline any potential self-selection bias or other biases that may be present and how these are likely to impact results.*
Ethics oversight	Massachusetts Institute of Technology Committee on the Use of Humans as Experimental Subjects (COUHES) approved this study does not involve human subjects as defined by federal regulations.

Note that full information on the approval of the study protocol must also be provided in the manuscript.

Field-specific reporting

Please select the one below that is the best fit for your research. If you are not sure, read the appropriate sections before making your selection.

☒ Life sciences ☐ Behavioural & social sciences ☐ Ecological, evolutionary & environmental sciences

For a reference copy of the document with all sections, see nature.com/documents/nr-reporting-summary-flat.pdf

Life sciences study design

All studies must disclose on these points even when the disclosure is negative.

Sample size	Due to the limited availability of human primary tissue samples, we initially performed genome-wide H3K27ac profiling in 517 human samples across brain, heart, muscle, and lung, with more than 100 samples for each tissue.
Data exclusions	After stringent quality control based on both standard ENCODE pipeline and tissue-specificity criterion, 387 profiles were kept for analyses.
Replication	Since very few data are available for haQTLs in brain, muscle, heart, and lung, we did not carry out replication analysis. Instead, we checked the sharing of haQTL effect among our four tissues, and observed consistency between tissues.
Randomization	During haQTL mapping, individuals were grouped based on genotypes, and covariates such as sex, age, and inferred latent factors were taken into consideration.
Blinding	Individuals are grouped based on genotypes, which is blind to investigators.

Reporting for specific materials, systems and methods

We require information from authors about some types of materials, experimental systems and methods used in many studies. Here, indicate whether each material, system or method listed is relevant to your study. If you are not sure if a list item applies to your research, read the appropriate section before selecting a response.

Materials & experimental systems

n/a	Involved in the study
☐	☒ Antibodies
☒	☐ Eukaryotic cell lines
☒	☐ Palaeontology and archaeology
☒	☐ Animals and other organisms
☒	☐ Clinical data
☒	☐ Dual use research of concern

Methods

n/a	Involved in the study
☐	☒ ChIP-seq
☒	☐ Flow cytometry
☒	☐ MRI-based neuroimaging

Antibodies

Antibodies used	Rabbit monoclonal antibody to the H3K27ac epitope (Cell Signaling Technology, Cat#8173), with 1:100 dilution.
Validation	This antibody has been validated by the manufacturer using SimpleChIP® Enzymatic Chromatin IP Kits. It was also validated in ENOCDE project (https://www.encodeproject.org/antibodies/ENCAB502OHI/), and has been cited in more than 300 other papers.

ChIP-seq

Data deposition

☒ Confirm that both raw and final processed data have been deposited in a public database such as GEO.

☒ Confirm that you have deposited or provided access to graph files (e.g. BED files) for the called peaks.

Data access links<br>*May remain private before publication.*	https://doi.org/10.5281/zenodo.7992724
Files in database submission	H3K27ac ChIP-seq profiles from this study are available on dbGaP with accession number phs000424.v8.p2. Additionally, the data can be accessed via AnVIL with authentication: https://anvil.terra.bio/#workspaces/anvil-datastorage/AnVIL_GTEx_V8_hg38; All non-protected data of H3K27ac ChIP-seq can be visualized via the GTEx Portal (www.gtexportal.org) as part of eGTEx v8. Proccessed data could be found on Zenodo (https://doi.org/10.5281/zenodo.7992724).
Genome browser session<br>(e.g. UCSC)	https://gtexportal.org/home/. Check IGV browser for detail.

Methodology

Replicates	We do not have biological replicates for each individual.
Sequencing depth	pair-end data, reads length 101bp. Please refer to supplementary Table 1 for the total reads of each of 387 experiments.
Antibodies	monoclonal antibody to the H3K27ac epitope (Cell Signaling Technology, #8173)
Peak calling parameters	macs2 callpeak -t ${REP1_TA_FILE}.tagAlign.gz -f BED -n ${PEAK_OUTPUT_DIR}/${CHIP_TA_PREFIX} -g ${GENOMESIZE} -p 1e-2 --nomodel --shift 0 --extsize ${FRAGLEN} --keep-dup all -B --SPMR
Data quality	Samples were QCed based on three metrics: (1) Relative Strand Cross-correlation coefficient (RSC), based on the (shifted) agreement between forward-strand and reverse-strand reads, providing a measure of signal-to-noise enrichment ratio; (2) sequencing depth, counted as total reads; (3) H3K27ac signal correlation between our samples and the samples from Roadmap epigenomes based on tissue-specific AREs identified from Roadmap. Please refer to Supplementary Table 1 for details, including number of peaks for each sample.
Software	Raw sequencing data was aligned and processed using bwa and picard, followed by AQUAS pipeline (https://github.com/kundajelab/chipseq_pipeline)
